# Pharmacists’ Knowledge, and Insights in Implementing Pharmacogenomics in Saudi Arabia

**DOI:** 10.3390/ijerph191610073

**Published:** 2022-08-15

**Authors:** Zahra Abdulathim Alhaddad, Hissah Abdullatif AlMousa, Nancy S. Younis

**Affiliations:** Department of Pharmaceutical Sciences, College of Clinical Pharmacy, King Faisal University, Al-Ahsa 31982, Saudi Arabia

**Keywords:** pharmacogenomics, pharmacists, education, self-confidence

## Abstract

Background: Pharmacogenomics (PGx) and personalized medicine embrace the potential to optimize drug treatment and improve the patient’s quality of life. Pharmacists’ roles include contributing to genetic testing, patient counseling, and pharmacotherapies selection for superior treatment outcomes. The aim of this study is to assess the pharmacists’ knowledge, insight, and self-confidence toward PGx testing, identify their future preferred education patterns, and determine the barriers to pharmacogenomic testing implementation. Method: A cross-sectional study was conducted using a previously validated questionnaire among pharmacists working in the Kingdom of Saudi Arabia (KSA). The questionnaire was designed in seven major categories, consisting of 26 questions. Results: A total of 671 pharmacists participated in this survey. As for knowledge, only 29.8% of pharmacists had good knowledge regarding PGx, while 42.9% had poor knowledge levels. Respectable PGx knowledge was significantly higher among outpatient dispensing pharmacists (33.6%; *p* = 0.049) and among pharmacists who had completed PGx testing-related training or education (40.3%; *p* = 0.001). Considering perception, it was positive among 50% of pharmacists and negative among 19.8%. With regard to self-confidence, it was high among 39.2% of male pharmacists (*p* = 0.042), among 43% of clinical pharmacists (*p* = 0.006), and among 44.8% of pharmacists who had extra credentials (*p* = 0.001). The utmost favored continuing-education learning approaches were workshops or seminars. The barriers to the implementation of PGx testing included a lack of testing devices, clinical guidelines, training or education, and personnel. Conclusion: The present study revealed that pharmacists in KSA had inadequate knowledge and understanding of PGx. Nevertheless, the majority established that PGx is a valuable tool for augmenting drug efficacy and safety.

## 1. Introduction

Pharmacogenetics is the study of the fluctuations in drug responses due to genetic differences [1]. It merges both pharmacology and genomics to develop an effective and safe drug that is specifically tailored to a person’s genetic makeup [2]. Pharmacogenomics (PGx) and personalized medicine embrace the potential to optimize drug treatment and improve the patients’ quality of life via diminishing adverse drug reactions and/or maximizing drug efficacy [3]. Personalized medicine is anticipated to revolutionize the practice of most medical approaches, offering more efficacious pharmacotherapy with the lowest possible side effects for each individual patient, based mainly on the patient’s genetic profiling [4]. Food and Drug Administration (US-FDA) has incorporated pharmacogenomics information details into more than 350 drug labels, specifically drugs with narrow therapeutic index and/or possibly toxic drugs such as chemotherapy [5], blood thinners [6], and anti-seizure [7] medications. From June 2019, 132 pharmacogenomic dosing guidelines are present for 99 drugs, and pharmacogenomic information is included in 309 medication labels. Additionally, guidelines for implementation of this data are available from organizations such as the Clinical Pharmacogenetics Implementation Consortium (CPIC) and the Dutch Pharmacogenetics Working Group (DPWG) [8,9]. Next-generation sequencing (NGS) technologies are evolving as a more comprehensive and time- and cost-effective [10] tool for genotyping patients for numerous pharmacogenomic loci. Nevertheless, the rate of applying genomic knowledge remains low in actual clinical settings, causing a gap in knowledge translated to patient care. Numerous aspects contribute to this translational gap between knowledge and clinical application, in which health care team education and decision support to apply pharmacogenomics-based decisions play an important role [11]. An integrative health care team, including physicians, nurses, genetic counselors, and pharmacists, is crucial to conveying pharmacogenomic-informed health care. All healthcare team members need to combine their expertise and knowledge to provide pharmacogenomic-informed clinical decisions [12]. Implementing pharmacogenomics testing approaches will alert physicians to prescribed medications with specific genetic variants that are concomitant with adverse drug reactions and/or drug effectiveness [13]. Another advantage of applying PGx is the reduction in the cost of the healthcare system via decreasing drug adverse reactions and failed trial numbers, as well as a faster drug approval process [14]. PGx training among pharmacists is being implemented in certain countries such as the USA and Europe, however, others are still behind in executing PGx training among pharmacists [15]. Pharmacists who have completed PGx training may contribute to enhancing medication compliance among patients via tailoring medications according to individual patient needs [16]. Pharmacists have the ability to impact all healthcare providers about the substantial importance of PGx in the healthcare system [17]. Genetic disorders are prevalent to a greater extent in KSA related to other Gulf or Arab countries because consanguinity marriage is habitually popular in the Kingdom [18]. Given the pharmacists’ dynamic roles in executing PGx testing, this study intended to evaluate pharmacists’ knowledge, insight, and self-confidence toward PGx testing; to recognize their preferred learning format for their future education; and finally, to identify the barriers to implementing pharmacogenomic testing knowledge in the clinical sites. 

## 2. Materials and Methods

### 2.1. Ethical Approval

The study was approved by the Ethical Conduct of Research in King Faisal University with the ethical approval number KFU-REC-2021-NOV-EA000140.

### 2.2. Study Design

A web-based survey was distributed online using Google Forms. This descriptive, cross-sectional survey was conducted between July and August 2021. The study included pharmacists working in the KSA by using a simple random method. Those who agreed to participate were provided with a detailed explanation of the study’s aim and were assured that participation was strictly voluntary and confidential. The sample size calculation was carried out based on the assumption that the response rate is 50%. The sample size was determined using the Raosoft sample-size calculator, providing a confidence interval of 95% and a margin of error of 5%. The target population was 1000 pharmacists currently practicing in KSA. The minimum sample size was estimated as 500 pharmacists. 

### 2.3. Study Tool 

To conduct this survey, a previously validated and published questionnaire was utilized [19]. The questionnaire consisted of 26 questions in seven major sections. The first section included questions 1–6 collected the participants’ demographic and professional characteristics and occupation, including the current level of practice and any extra credentials apart from a pharmacy bachelor’s degree. The second section consisted of three questions about PGx training/education and application in practice (questions 7–10). The third section included five questions (questions 11–14) and assessed the pharmacists’ perceptions of the common knowledge on PGx and its consequence. The fourth segment of the questionnaire included six questions (questions 15–20) regarding the perceptions of pharmacists towards pharmacogenetics and its implications. Afterward, the questionnaire moved on to the fifth segment, which evaluated the pharmacists’ confidence in applying PGx in practice settings. The sixth section was about the pharmacist’s preference for the future pharmacogenetics education mode. The last section of the questionnaire included the obstacles to the application of pharmacogenetics testing in different practice settings. 

### 2.4. Data Analysis

After data were collected, modified, coded, and entered into statistical software (IBM SPSS version 22, SPSS, Inc., Chicago, IL, USA). All statistical analysis was done using two-tailed tests. A *p*-value less than 0.05 was considered to be statistically significant. In the analysis of the third section regarding pharmacists’ knowledge of PGx and its consequences, a score of 1 was given for each correct answer, or zero for each question answered wrong. Thus, a maximum score of 5 can be achieved by correctly answering all the knowledge-based questions. A score of 4.0 or higher was considered good knowledge, a score < 4.0 or higher than 2.5 was considered moderate knowledge, and a score of 2.5 or less was considered poor knowledge. A 3-point Likert scale measured responses to both perception and self-confidence-based questions (i.e., agree, neutral, or disagree). As for the pharmacists’ perceptions, a score of 2.0 was given for each time a participant answered “agree”, 1.0 for “neutral”, and 0.0 for “disagree”. Thus, the maximum score a participant could achieve is by answering “agree” to all of the perception questions was 12. The total mean score of all the participants was calculated, and a total mean score of 10.0 and higher was considered a positive perception. On the other hand, a total mean score of 6.0 or less was considered a negative perception. For the self-confidence-based questions, a score of 2.0 was given for each time a participant answered “agree”, 1.0 for “neutral”, and 0.0 for “disagree”. Thus, the maximum score a participant could achieve by answering “agree” to all of the self-confidence-based questions was 8.0. The total mean score of all participants was calculated, and a total mean score of 6.0 or more was considered high. On the other hand, a total mean score of 4.0 or less was considered low. A descriptive analysis based on frequency and percent distribution was done for all variables, including pharmacists’ personal data, knowledge, perception, and self-confidence, which were tabulated and graphed. In addition, barriers to the implementation of pharmacogenetic testing and the type of education preferred by study pharmacists were graphed. Cross tabulation was used to assess factors associated with pharmacists’ knowledge, perception, and self-confidence of PGx. Pearson chi-square test with exact tests were used to assess statistical significance for all relations due to small frequency distribution.

## 3. Results

### 3.1. Demographic Characteristics and Professional Information of the Study Participants

Around 671 pharmacists participated, of which 277 (41.2%) were from the central area, 137 (20.4%) from the western area, 130 (19.3%) from the eastern area, 65 (9.7%) from the northern area, and 63 (9.4%) from the southern area. Participants’ ages ranged from 24 to 60 years old, with a mean age of 29.4 ± 13.5 years old. Exactly 385 (57.3%) pharmacists were males. As for experience years, it was one year among 174 (25.9%), 1–3 years among 210 (31.3%), and 4–6 among 121 (18%). A total of 229 (34.1%) were outpatient dispensing pharmacists, 140 (20.8%) were inpatient dispensing pharmacists, and 128 (19%) were clinical pharmacists. A total of 241 (35.9%) had extra credentials (postgraduate study) apart from a bachelor’s degree, and 226 (33.6%) completed PGx testing-related training or education. Out of the 671 pharmacists who participated, only 152 (22.6%) applied pharmacogenetic testing to drug therapy selection, dosing, and monitoring in the practice setting, and merely 145 (21.6%) counseled patients on the results of the pharmacogenomic testing (Table 1). 

### 3.2. Pharmacogenetics General Knowledge among the Study Participants

In this section, various questions were used to evaluate the knowledge of pharmacogenomics among pharmacists who participated in this study. Among the participants, only 17.9% correctly answered the question on whether the genetic determinants of drug response change over a person’s lifetime. Whereas 330 (49.3%) responded with the wrong answer. Regarding the availability of pharmacogenetics testing for most medications, only 171 (25.6%) knew it was available, whereas 225 (33.7%) thought that PGx testing was unavailable. Additionally, 349 (52.2%) of the responders knew that the warfarin package insert includes a warning about altered metabolism in individuals who have specific genetic variants. As for PGx’s essential role in individualizing response to medications, 482 participants (72.2%) knew the essential role of pharmacogenetics in individualizing response to medications. Concerning the role of PGx in identifying drug-drug interactions, 421 (63.3%) knew that it is crucial in identifying drug–drug interactions, as shown in Table 2. 

**Figure 1:**
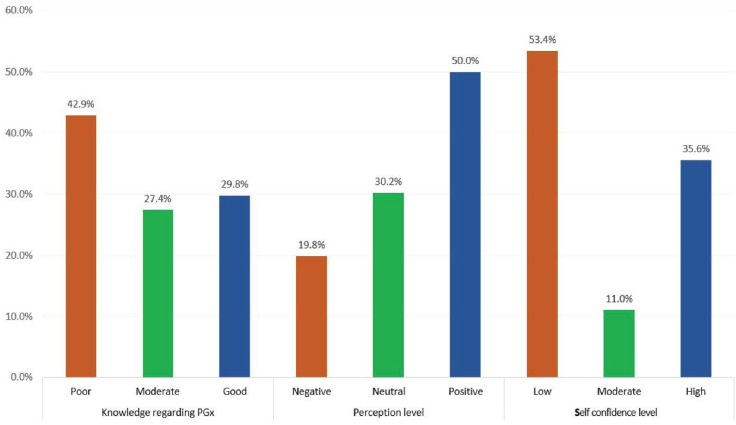
Knowledge, perception, and self-confidence among pharmacists working in KSA regarding PGx.

By applying the scoring system, the current study showed that most of the participants (49.2%) were poor general knowledge regarding PGx, as shown in Figure 1.

### 3.3. Participants’ Perceptions toward Pharmacogenetics and Its Implications

In order to assess the participants’ perceptions toward pharmacogenetics and its implications, some questions were asked in this questionnaire, as demonstrated in Table 3. Among the participants, 232 (34.7%) agreed that PGx is relevant to their current clinical practice and 375 (56.1%) believed that pharmacogenetics testing should be applied to their clinical practice. Moreover, 466 (69.7%) agreed that pharmacogenetics would improve their ability to control drug therapy expenditures more efficiently. Of the pharmacists who participated in the current study, 487 (72.7%) believed that pharmacists should be required to have some knowledge of pharmacogenetics. Regarding the role of the pharmacists, 400 (59.7%) assumed that the pharmacists should be able to provide information on the appropriate use of pharmacogenetics testing, as clarified in Table 3.

### 3.4. Participants’ Self-Confidence in Applying Pharmacogenetics in Their Practice Settings

A total of 241 (36.1%) and 239 (35.9%) acknowledged that they could confidently identify drugs that need PGx testing and reliable sources of information regarding PGx testing for healthcare professionals and patients, respectively. On the other hand, only 250 (37.3%) participants felt confident in applying the results of PGx testing to drug therapy selection and dosing accurately or monitoring, as shown in Table 4. In addition, 188 (28.1%) participants confidently determined the availability of PGx testing within the Saudi healthcare system.

### 3.5. Factors Associated with Pharmacists’ Knowledge, Perception and Self-Confidence of PGx in Saudi Arabia

As for pharmacists’ knowledge, good knowledge was significantly higher among pharmacists who had completed PGx testing-related training or education (40.3%, *p* = 0.001) and among the pharmacists who counseled patients on the results of their pharmacogenomic testing (42.1%, *p* = 0.001). Considering pharmacists’ perception, it was positive among young-aged pharmacists (51.5%, *p* = 0.043) and among clinical pharmacists (55.5%, *p* = 0.004). With regard to self-confidence, it was high among male pharmacists (39.2%, *p* = 0.042), among clinical pharmacists (43%, *p* = 0.006), and among the pharmacists who had extra credentials (44.8%, *p* = 0.001). Additionally, self-confidence was high among pharmacists who completed PGx testing-related training or education (43.4%, *p* = 0.001), 56.6% of pharmacists who applied pharmacogenetic testing to drug therapy selection, dosing, and monitoring (*p* = 0.001), and 57.9% of those who counseled patients on the results of their pharmacogenomics testing (*p* = 0.001), as illustrated in Table 4 and Figure 1. As for knowledge, 200 (29.8%) pharmacists had good knowledge regarding PGx, while 288 (42.9%) had a poor knowledge level. Considering perception, it was positive among 336 (50%) pharmacists and negative among 133 (19.8%). Regarding self-confidence, it was high among 239 (35.6%) pharmacists and low among 359 (53.4%) of them, as shown in Figure 1.

### 3.6. Participants Preferred Continuing-Education Program Learning Styles and the Challenges They Face in Implementing Pharmacogenetics Testing in Their Clinical Practice

**Figure 2:**
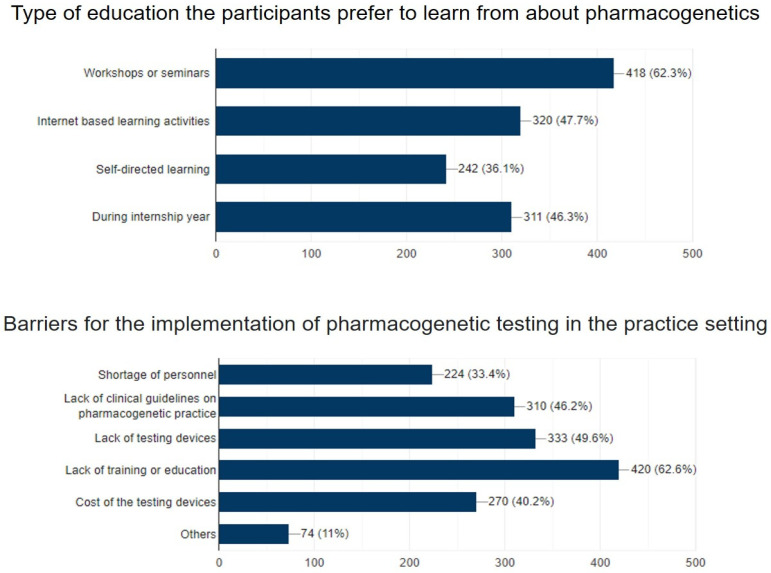
Participants preferred continuing-education program learning styles and the challenges the pharmacists face in implementing pharmacogenetic testing in their clinical practice.

Regarding preferred continuing-education program learning styles, the utmost favored continuing-education learning approaches were workshops or seminars 62.3%, followed by internet-based learning activities 47.7%, during the internship year 46.3%, and self-directed learning 36.1%. The last section of the questionnaire included the barriers to the application of pharmacogenetic testing, in which we tried to identify barriers to the implementation of pharmacogenetic testing in their practice setting from the pharmacists’ point of view. The most reported barriers were lack of training or education (62.6%), followed by lack of testing devices (49.6%), lack of clinical guidelines on PGx practice (46.2%), cost of the testing devices (40.2%), shortage of personnel (33.4%), and cost of the testing devices (4%), as illustrated in Figure 2.

## 4. Discussion

PGx testing practice is minimal in KSA and only occurs in certain specialist cancer centers with specific medications. Due to the influential role of pharmacists in the clinical implementation of PGx testing, the current study was designed to conclude their knowledge, insights, and self-confidence in the clinical implementation of PGx testing in their clinical practices in KSA. Of the participating pharmacists, the majority have not completed any PGx testing-related training or education. Subsequently, the majority of the participants have not applied any PGx testing to drug therapy selection, dosing, and monitoring for any of their patients, nor have they performed any patient counseling on PGx testing results, which are imperative parts of the pharmacists’ role. 

Five varied questions were utilized to assess the pharmacogenomics knowledge among the pharmacists who participated in this existing study, and the results clearly distinguished that pharmacists have inadequate knowledge and understanding of PGx and its role in optimizing drug therapy and minimizing adverse drug reactions. A similar survey was conducted in Kuwait [2], which showed that pharmacists in KSA as well as those working in Kuwait both had similar limited or partial understanding regarding PGx testing. For instance, only 16.2% of the hospital pharmacists in Kuwait, compared to 17.8% of pharmacists in KSA, recognized that genetic determinants of drug responses do not change over a person’s lifetime. On the other hand, another survey was performed in Jeddah, which showed a much higher level, reaching 70.6%, which may be due to the fact that all the participants in the Jeddah questionnaire were only hospital pharmacists [19]. 

The next section of the questionnaire was designed to assess the participants’ insights toward pharmacogenetics and its implications. Only 34.7% of the participating pharmacists decided that PGx was relevant to their current clinical practice. However, the majority believed that pharmacists should be required to have some knowledge of pharmacogenetics and that it should be implemented into their clinical practice. These results clearly indicate the deficiency of knowledge regarding PGx testing, which could be associated with the fact that the PGx course is not incorporated into the undergraduate pharmacy program curricula in KSA. A minority of medical schools in Europe and North America already include pharmacogenomics as part of their core pharmacology curricula [4]. Furthermore, the PGx course has been integrated into the curricula of most colleges of pharmacy since 2007 in the USA as a required competency for pharmacy education accreditation. At the moment, there are more than twenty governmental pharmacy colleges and at least two private colleges in Saudi Arabia that offer PharmD programs [20]. An essential step forward would be to incorporate PGx into these PharmD programs’ curricula as a full course for an academic semester, which will provide PharmD graduates with the essential information about PGx testing beforehand performing it clinically.

The lack of self-confidence among pharmacists is an additional major challenge in applying PGx testing. Only 36.1% felt confident in identifying drugs that need PGx testing, applying PGx testing results to drug therapy selection and dosing, and identifying reliable sources of information regarding PGx testing for healthcare professionals and patients. In harmony with earlier studies, the witnessed lack of self-confidence among pharmacists has been demonstrated not only in KSA [19] but also in several places such as Kuwait [2], USA [21], and others [22]. Pharmacists’ lack of self-confidence in KSA could be attributed to the lack of adequate education and training. This is why PGx courses for undergraduate students and continuing education and training for licensed pharmacists are imperative to increase pharmacists’ self-confidence to perform PGx-based clinical decisions. 

Regarding the preferred continuing-education program learning styles by the participating pharmacists, the utmost favored continuing-education learning approaches were workshops or seminars, followed by internet-based learning activities during the internship year and self-directed learning. KSA has an obligatory continuing education system (CME) for pharmacists to continue their licenses as the pharmacists need to complete 20 h CME annually to maintain the pharmacy license [23]. Continuing education programs will likely increase pharmacists’ knowledge and self-confidence about PGx, which is essential for the appropriate delivery of PGx testing. Thus, the results of the current study strongly highlight the significance of CME as a tool to assist in educating, training, and implementing PGx into clinical practice in KSA. In agreement with our results, several studies reported pharmacists’ willingness to implement PGx testing to assist physicians in making appropriate treatment decisions [24,25]. 

The barriers to the application of pharmacogenetic testing were the lack of testing devices, which was the greatest barrier, followed by lack of clinical guidelines on pharmacogenetics practice, lack of training or education, and shortage of personnel. Whereas the smallest obstacle that pharmacists are facing in KSA for the implementation of pharmacogenetics testing in their practice setting was the cost of the testing devices. Klein and Parvez [14] published an extensive review on the ongoing clinical implementation programs and identified several scientific, educational, ethical, legal, and social issues, information technology, and reimbursement as the key barriers to PGx application clinically. The key solutions which Klein and Parvez [14] suggested for these barriers include the construction of a secure and suitable information technology infrastructure combined with clinical decision support schemes, along with expanding PGx evidence, more procedures, reimbursement approaches for stakeholder recognition, integration of PGx teaching in all institutes and clinics, and PGx promotion to health care professionals.

Limitations of the study: An integrative health care team, including physicians, nurses, genetic counselors, and pharmacists, is crucial to delivering efficient pharmacogenomic-informed clinical decisions. In this study, we only focused on pharmacists, not the remaining healthcare members, and consequently, this is one of the limitations of this study. Another limitation of the current study is that it is not specifically examining the role of pharmacists with regard to the implementation of pharmacogenomics as stated in ASHP.

## 5. Conclusions

The present study revealed that pharmacists in KSA had limited knowledge and understanding of PGx testing. Nevertheless, the majority expressed a high level of awareness and established that PGx testing is a valuable tool for augmenting drug efficacy and safety. The study also emphasized the significance of providing appropriate informative resources on PGx for pharmacists to deliver PGx consultations effectively. PGx incorporation will definitely increase treatment efficacy as well as diminish adverse event risks, which will improve the future of the patient care system as a whole.

## Figures and Tables

**Table 1:** Demographic characteristics and professional information of the participants (*n* = 671).

Personal Data	No	%
**Region**		
Central area	277	41.2%
East area	130	19.3%
North Area	65	9.7%
Southern area	63	9.4%
West area	137	20.4%
**Age in years**		
24–35	532	79.2%
36–45	108	16.1%
46+	32	4.8%
**Gender**		
Male	385	57.3%
Female	287	42.7%
**Professional experience (in years)**		
0–1	174	25.9%
1–3	210	31.3%
4–6	121	18.0%
7–9	53	7.9%
>9	114	17.0%
**What is your profession and current level of practice?**		
Clinical pharmacist	128	19.0%
Inpatient dispensing pharmacist	140	20.8%
Others	175	26.0%
Outpatient dispensing pharmacist	229	34.1%
**Do you have any extra credentials (postgraduate study) apart from your bachelor’s degree?**		
Yes	241	35.9%
No	431	64.1%
**Have you completed pharmacogenetic testing related training or education?**		
Yes	226	33.6%
No	446	66.4%
**Have you applied pharmacogenetic testing to drug therapy selection, dosing and monitoring for a patient in your practice setting?**		
Yes	152	22.6%
No	520	77.4%
**Have you counselled patients on the results of their pharmacogenomics testing in your practice setting?**		
Yes	145	21.6%
No	527	78.4%

**Table 2:** Participant’s knowledge on pharmacogenetics.

General Knowledge of Pharmacogenetics	Correct Answer	Answering the Correct Answer Which Is False *n* (%)	Answering the Wrong Answer Which Is True *n* (%)	Answering the Wrong Answer Which Is Do Not Know/Not Sure *n* (%)
Genetic determinants of drug response change over a person’s lifetime.	False	119 (17.8%)	330 (49.3%)	220 (32.9%)
The package insert for warfarin includes a warning about altered metabolism in individuals who have specific genetic variants.	True	73 (10.9%)	349 (52.2%)	247 (36.9%)
Pharmacogenetics testing is currently available for most medications.	False	225 (33.7%)	171 (25.6%)	271 (40.6%)
Pharmacogenetics has an important role in individualizing response to medications.	True	55 (8.2%)	482 (72.2%)	131 (19.6%)
Pharmacogenetics has an important role in identifying drug-drug interactions.	True	91 (13.6%)	421 (63.3%)	131 (19.6%)

**Table 3:** Participants’ perceptions and self-confidence toward pharmacogenetics, its implications, and training.

Variable		Number of Participants *n* (%)
**Pharmacogenetics training/education and application in practice**		
Completed any pharmacogenetics testing related training or education.	No Yes	445 (66.3%) 226 (33.7%)
Applied any pharmacogenetics testing to drug therapy selection, dosing, and monitoring for a patient in their practice setting.	No Yes	519 (77.3%) 152 (22.7%)
Counseled patients on the results of their pharmacogenomics testing.	No Yes	526 (78.4%) 145 (21.6%)
**Perceptions towards pharmacogenetics and its implications**		
Pharmacogenetics is relevant to my current clinical practice.	Agree Neutral Disagree	232 (34.7%) 285 (42.6%) 152 (22.7%)
Pharmacists should be required to have some knowledge of pharmacogenetics.	Agree Neutral Disagree	487 (72.7%) 151 (22.5%) 32 (4.8%)
Pharmacogenetic testing should be applied into my clinical practice.	Agree Neutral Disagree	375 (56.1%) 228 (34.1%) 66 (9.9%)
Pharmacists should be asked by healthcare professionals for recommendations on appropriate use of pharmacogenetic testing.	Agree Neutral Disagree	405 (60.5%) 222 (33.2%) 42 (6.3%)
I should be able to provide information on appropriate use of pharmacogenetic testing.	Agree Neutral Disagree	400 (59.7%) 208 (31%) 62 (9.3%)
Pharmacogenetics will improve our ability to more effectively control drug therapy expenditures.	Agree Neutral Disagree	466 (69.7%) 158 (23.6%) 45 (6.7%)
**Confidence in applying pharmacogenetics in your practice settings**		
I can identify drugs that need pharmacogenetic testing.	Agree Neutral Disagree	241 (36.1%) 285 (42.7%) 142 (21.3%)
I can identify reliable sources of information regarding pharmacogenetics for healthcare professionals and patients.	Agree Neutral Disagree	239 (35.9%) 295 (44.3%) 132 (19.8%)
I can readily determine the available pharmacogenetic tests within our healthcare system.	Agree Neutral Disagree	188 (28.1%) 288 (43%) 193 (28.8%)
I can accurately apply the results of a pharmacogenetic test to drug therapy selection, dosing, or monitoring	Agree Neutral Disagree	250 (37.3%) 274 (40.9%) 146 (21.8%)

**Table 4:** Factors associated with pharmacists’ knowledge, perception, and self-confidence of PGx in Saudi Arabia.

	Good Knowledge		Positive Perception		High Self-Confidence	
	No	%	No	%	No	%
**Age in years**						
24–35	163	30.6%	274	51.5%	188	35.3%
36–45	29	26.9%	54	50.0%	39	36.1%
46+	8	25.0%	8	25.0%	12	37.5%
*p*-value	0.228		0.043 *		0.167	
**Gender**						
Male	125	32.5%	181	47.0%	151	39.2%
Female	75	26.1%	155	54.0%	88	30.7%
*p*-value	0.139		0.140		0.042 *	
**Professional experience (in years)**						
<6 years	155	30.7%	244	48.3%	173	34.3%
>6 years	45	26.9%	92	55.1%	66	39.5%
*p*-value	0.569		0.249		0.457	
**What is your profession and current level of practice?**						
Clinical pharmacist	40	31.3%	71	55.5%	55	43.0%
Inpatient dispensing pharmacist	30	21.4%	53	37.9%	36	25.7%
Others	53	30.3%	93	53.1%	55	31.4%
Outpatient dispensing pharmacist	77	33.6%	119	52.0%	93	40.6%
*p*-value	0.049 *		0.004 *		0.006 *	
**Do you have any extra credentials (postgraduate study) apart from your bachelor’s degree?**						
Yes	80	33.2%	126	52.3%	108	44.8%
No	120	27.8%	210	48.7%	131	30.4%
*p*-value	0.245		0.214		0.001 *	
**Have you completed pharmacogenetic testing related training or education?**						
Yes	91	40.3%	122	54.0%	98	43.4%
No	109	24.4%	214	48.0%	141	31.6%
*p*-value	0.001 *		0.081		0.001 *	
**Have you applied pharmacogenetic testing to drug therapy selection, dosing and monitoring for a patient in your practice setting?**						
Yes	54	35.5%	73	48.0%	86	56.6%
No	146	28.1%	263	50.6%	153	29.4%
*p*-value	0.185		0.856		0.001 *	
**Have you counselled patients on the results of their pharmacogenomic testing in your practice setting?**						
Yes	61	42.1%	74	51.0%	84	57.9%
No	139	26.4%	262	49.7%	155	29.4%
*p*-value	0.001 *		0.674		0.001 *	

* *p* < 0.05 (significant).
